# Methylomic Changes in MTHFR Promoter Region, along with the Heterozygous C677T Polymorphism, Contribute to the Risk of Thrombotic Stroke

**DOI:** 10.1007/s12031-025-02364-1

**Published:** 2025-07-21

**Authors:** Ahmed M. Zain, Khalil A. El-Halfaway, Ahmed A. Abdel Megeed, Ahmed Abd Elbadee, Hany Khalil

**Affiliations:** 1 Department of Molecular Biology, Genetic Engineering and Biotechnology Research Institute, University of Sadat City, Menoufia Governate, El-Sadat, Egypt; 2 Clinical Pathology, International Medical Center, Cario Governate, El Shorouk, Egypt; 3 Department of Clinical Pathology, Milaitry Medical Acadmy, Cairo, Egypt; 4 Animal Biotechnology Department, Genetic Engineering and Biotechnology Research Institute, University of Sadat City, MenoufiaGovernate, El-Sadat, Egypt

**Keywords:** Thrombotic strokes, SNPs, Methylation activates, FV, FII, MTHFR

## Abstract

Stroke is the second leading cause of death globally and a major contributor to disability. Developing countries report the highest rates of stroke, with ischemic stroke being the most prevalent type. This study aimed to explore the potential association between specific single nucleotide polymorphisms (SNPs) and thrombotic strokes in Egyptian patients, as well as the role of DNA methylation in the promoter regions of genes associated with these SNPs. The study involved 100 adult patients who were consecutively admitted to the International Medical Center. These patients, diagnosed with acute ischemic stroke, were compared to age-matched control subjects (± 3 years). Molecular analysis was conducted on six thrombosis-related SNPs: FV (R506Q, H1299R, Y1702C), FII (G20210A), and MTHFR (C677T, A1298C) using blood samples from both stroke patients and healthy controls. DNA methylation in the promoter regions of the FV, FII, and MTHFR genes was assessed through a sodium bisulfite conversion protocol and genomic DNA digestion with the methylation-dependent restriction enzyme MspJI, using specific primers for the promoter regions of FV, FII, and MTHFR in all derived samples. The biochemical analysis of the derived samples revealed elevated levels of homocysteine, ESR, and LDL in stroke patients, alongside reduced levels of both vitamin B12 and serum folate. The SNP analysis of samples from healthy controls and stroke patients, conducted using the TaqMan™ SNP genotyping assay, identified the homozygous SNPs in the FV, FII, and MTHFR genes. The results clearly show that the MTHFR C677T heterozygous mutation is present in nearly all stroke patient samples, with a very low likelihood of this mutation co-occurring with SNP mutations in the other indicated genes. Analysis of methylation activities in the promoter regions of the indicated genes showed hypermethylation in the MTHFR promoter region, while methylation levels in the FV and FII promoter regions were normal. The analysis showed increased methylation of cytosine nucleotide in the MTHFR promoter region, potentially inhibiting MTHFR expression and contributing to the development of thrombotic strokes in patients. Overall, the data support an association between the MTHFR C677T mutation, hypermethylation in its promoter region, and stroke development in the study participants.

## Introduction

Stroke is a clearly defined clinical syndrome characterized by a sudden, localized neurological deficit resulting from a vascular injury to the central nervous system. It ranks as the second leading cause of death and disability globally. Stroke is not a singular condition; it can be triggered by various risk factors, underlying diseases, and mechanisms (Kuriakose and Xiao 2020). Strokes can be broadly categorized into two main types: ischemic and hemorrhagic. Ischemic strokes, which account for about 85% of all stroke cases, occur when a blood vessel in the brain is blocked, often by a clot. Hemorrhagic strokes, caused by bleeding, make up roughly 15% of strokes (Murphy and Werring 2020). The ischemic stroke happens when a blood vessel supplying the brain becomes obstructed, leading to a disruption in blood flow to a part of the brain. This lack of blood supply causes brain cells to die rapidly due to insufficient oxygen and nutrients (Barthels and Das 2020).

Ischemic strokes can be further divided into two types: thrombotic strokes, which result from a clot forming in a brain blood vessel, and embolic strokes, which occur when a clot or plaque debris forms elsewhere in the body and travels to the brain through the bloodstream (Gupta and Wagh 2023). The thrombotic stroke, a clot develops within one artery suppled the blood to the brain, blocking blood flow to a specific brain region (Griffin et al. 2016). This blockage typically forms in an artery already narrowed by atherosclerosis, a condition where fatty plaques accumulate within brain capillaries. Thrombotic strokes can affect both large and small brain arteries. When large arteries are affected, the blockage disrupts blood flow to a larger portion of the brain, often resulting in more severe disability. Instead, when the stroke occurs in a small artery, especially deep in the brain, it is known as a lacunar stroke (Rojsanga et al. 2019). These strokes generally cause fewer symptoms, as they only impact a smaller area of the brain. The primary focus of stroke treatment is to restore supplied blood to the brain, in addition to mitigate the neurological damage caused by the stroke (Paul and Candelario-Jalil 2021).

Single nucleotide polymorphisms (SNPs) are valuable markers and tools for studying population structures, conducting linkage analysis, performing genome-wide association studies, and facilitating breeding and population management. However, the availability of SNP markers has been primarily restricted to the most commercially significant timber species, mainly due to the high costs associated with genome sequencing for SNP discovery (Ousmael et al. 2023). Several SNPs have been associated with an increased risk of venous thromboembolism (VTE), including those in the GP6 (rs1613662), SERPINC1 (rs2227589), F11 (rs2036914 and rs2289252), FGG (rs2066865), and F12 (rs1801020) genes. Specifically, the CC genotype of rs2036914 and the CT and TT genotypes of rs2289252 in the F11 gene were linked to a significantly higher risk of VTE. A trend suggesting a thrombogenic effect was also observed for the risk alleles in the GP6 and FGG SNPs (El-Galaly et al. 2013). The diverse clinical phenotypes and varying thrombosis manifestations seen in thrombophilic families suggest that susceptibility to venous thrombosis arises from the interplay of multiple genetic factors, including the Leiden mutation in the factor V gene (FV R506Q) and the prothrombin (PT) 20210G/A mutation (Castoldi et al. 2000). Raised homocysteine levels are a separate risk factor for cardiovascular diseases, ischemic stroke, and thrombosis.

The methylenetetrahydrofolate reductase (MTHFR) enzyme plays a crucial role in controlling homocysteine levels. Therefore, a C677T mutation in this gene results in decreased enzyme activity, which subsequently raises the risk of stroke (Alluri et al. 2005). Notably, homocysteine, a sulfur-containing amino acid, is produced during various transmethylation reactions that consume S-adenosyl methionine (SAM). It is converted back into methionine through remethylation by the vitamin B12-dependent enzyme methionine synthase. This process utilizes 5-methyltetrahydrofolate as the methyl donor. 5-Methyltetrahydrofolate is formed from 5,10-methylenetetrahydrofolate through the action of the MTHFR enzyme. Individuals who are homozygous or heterozygous for the C to T substitution at nucleotide 677 in the MTHFR gene experience a 50% decrease in enzyme activity, making this the most common inherited cause of moderate hyperhomocysteinemia (Frosst et al. 1995; Alluri et al. 2005).

DNA methylation, a key epigenetic modification, has recently been linked to several medical conditions and diseases, such as cancer, acute infections, cardiovascular diseases, neurodegenerative disorders, and stroke (Ilango et al. 2020; Khalil et al. 2022, 2024). This process involves the addition of a methyl group to the fifth carbon of cytosine, forming 5-methylcytosine. DNA methylation regulates gene expression by recruiting proteins that suppress gene activity or by blocking the binding of transcription factors to the DNA. Through development, the genome’s DNA methylation pattern changes dynamically through processes of de novo methylation and demethylation. Consequently, differentiated cells establish a stable and tissue-specific DNA methylation pattern that controls gene expression unique to each tissue type (Moore et al. 2013).

In this study, we explored the relationship between six thrombosis-related SNPs; FV (R506Q, H1299R, Y1702C), FII (G20210A), and MTHFR (C677T, A1298 C) and thrombotic strokes in Egyptian patients. Additionally, we examined the potential role of epigenetic modifications in the promoter regions of these genes, and their association with elevated homocysteine levels and vitamin B12 levels in patients with thrombotic strokes.

## Material and Methods

### Ethical Issues and Sample Collection

This study was conducted by the Genetic Engineering and Biotechnology Research Institute at the University of Sadat City, Egypt, in collaboration with the International Medical Center, from February to December 2024. The research received approval from both the medical ethics committee of the International Medical Center and the scientific ethics committee of the Genetic Engineering and Biotechnology Research Institute. The purpose of the study was thoroughly explained to all participants, who provided informed consent and understood the study’s rules and regulations. Key inclusion criteria were that samples were collected from both patients and healthy individuals with a mean age of 45 ± 10 years, prior to any exposure treatment. All patients were examined by a qualified stroke neurologist, and ischemic stroke was confirmed using CT scans and MRI. The exclusion criteria eliminated individuals with significant cardiac conditions, those younger than 35 or older than 55 years, and individuals with obesity, diabetes mellitus, tumors, renal or hepatic failure, or those taking medications that could influence levels of vitamin B12, folate, or zinc. Patients with thyroid or rheumatologic diseases, as well as those with a history of immunosuppressive or analgesic therapy, were also excluded. All participants underwent a comprehensive physical examination and standard biochemical testing, including a blood lipid profile. Clinical data collected from patients included hypertension, diabetes status, level of consciousness, swallowing difficulties, and smoking history. Control samples were collected from healthy individuals, comprising 50 males and 50 females within the study’s age range. These individuals had no history of clinical or radiological signs of cerebrovascular disease. Additionally, individuals who were smokers, underweight, suffering from cachexia, or had a history of eating disorders were excluded. A total of 100 blood samples were collected from patients diagnosed with thrombotic strokes, comprising 50 males and 50 females within the study’s specified age range. All stroke patients were accurately diagnosed and received medical care and supervision at the International Medical Center.

### Homocysteine Determination

The quantitative determination of homocysteine levels in plasma samples was performed using a human ELISA Kit (CSB-E13814 h, CUSABIO, USA), which contains a specific antibody pre-coated onto a microplate. Plasma samples were collected using EDTA as an anticoagulant, following the manufacturer's protocol. The samples were centrifuged within 30 min of collection at 1000 × g for 15 min at 2–8 °C. The plasma was then aliquoted and stored at −20 °C for up to 5 days before testing. Prior to testing, all reagents were allowed to equilibrate at room temperature for 30 min, and the standard solution was freshly prepared through serial dilution. A total of 100 µL of both the standards and samples were carefully pipetted into the wells of the ELISA plate. The plate was covered with the provided adhesive strip and incubated for 2 h at 37 °C. Afterward, 100 µL of biotin-labeled antibody (1x) was added to each well and mixed gently. The plate was covered again with a new adhesive strip and incubated for an additional hour at 37 °C. Following incubation, the buffer was removed from each well, and the plate was washed twice with 200 µL of Wash Buffer. Then, 100 µL of HRP-avidin (1x) was added to each well, and the plate was covered with a fresh adhesive strip and incubated for 1 h at 37 °C. After incubation, the plate was washed twice as described previously. Finally, 50 µL of stop solution was added to each well, and the optical density was measured within 5 min using an ELISA reader set to 450 nm.

### Erythrocyte Sedimentation Rate (ESR)

The Global Scientific Sedi-Rate™ (Autozero Westergren ESR System, USA) was used to measure the ESR in the collected samples. This system features a fibrous plug that prevents harmful substances from escaping through the top of the pipette. The polypropylene vial has a self-sealing stopper that can be easily pierced with a transfer pipette or piercing funnel, and the polystyrene pipette is graduated from 0 to 180 mm. To conduct the test, blood samples were drawn into a Westergren tube until the blood level reached 200 mm (mm). The tubes were then kept in a vertical position and stored at room temperature for one hour. The ESR was calculated by measuring the distance between the top of the blood mixture and the top of the sedimented red blood cells. The result, expressed in millimeters per hour (mm/h), indicates how quickly the red blood cells settle to the bottom of the test tube.

### Low-Density Lipoprotein (LDL) Measurement

The LDL concentration in the obtained samples was determined using Beta-quantification analysis. Plasma aliquots with a density of 1.006 g/mL were subjected to ultracentrifugation, which resulted in the separation of VLDL and chylomicrons in the floating layer (d < 1.006 g/mL) above the infranatant containing IDL, LDL, and HDL (d > 1.006 g/mL). Cholesterol concentration in the bottom fraction was then measured after treating it with heparin and manganese chloride to precipitate ApoB, including IDL and LDL particles. Following centrifugation, the supernatant was discarded, and the HDL cholesterol concentration in the bottom fraction was determined. The LDL cholesterol concentration was calculated by subtracting the HDL concentration from the total cholesterol concentration in the bottom fraction containing IDL, LDL, and HDL (d > 1.006 g/mL). The concentration of VLDL-C in the ultracentrifugal supernatant was measured by subtracting the cholesterol concentration in the bottom fraction (d > 1.006 g/mL) from the total cholesterol concentration in the initial plasma aliquot before ultracentrifugation. VLDL-C = Total cholesterol – d > 1.006 g/mL cholesterol. It is preferred to calculate VLDL-C rather than directly measure it in the ultracentrifugal supernatant, as recovering cholesterol from this fraction, particularly with a high concentration of triglycerides, is challenging. The LDL-C concentration was calculated as [d > 1.006 g/mL cholesterol] – HDL cholesterol. In blood obtained samples, the cut-off level of LDL-C was considered as 70 mg/dL (Khalil et al. 2019).

### Determination of Vitamin B12 and Folic Acid Levels

To measure vitamin B12 levels, 15 μL of serum was incubated for 9 min at 37 °C with vitamin B12 pretreatment reagents (pretreatment one and pretreatment two). Following this, the pretreated samples were incubated for another 9 min at 37 °C with a diluted (1:1000) solution of ruthenium-labeled vitamin B12 binding factor (REF 07212771190, Roche, Switzerland). This was followed by a further incubation for 9 min at 37 °C with diluted (1:1000) streptavidin-coated microparticles and biotin-labeled vitamin B12 (REF 07212771190, Roche, Switzerland). The reaction was then transferred into measuring cells, and the chemiluminescent emission generated was measured by a photomultiplier. The final values were calculated using a master curve provided via the reagent barcode. Similarly, folate levels in prepared serum were measured by incubating 25 μL of serum with folate pretreatment reagents (pretreatment one and two), a diluted (1:1000) solution of ruthenium-labeled folate binding protein, and biotin-labeled folate (REF 07559992190, Roche, Switzerland). Deficiency was defined as vitamin B12 levels < 203 pg/ml and folate levels < 3 ng/ml (ÖZKAN et al. 2007; Singh et al. 2017).

### SNP Analysis in Stroke Patients Derived Blood Samples

The genotyping of six thrombosis-related SNPs (FV R506Q, H1299R, Y1702 C, FII G20210 A, and MTHFR C677 T, A1298 C) in blood samples from stroke patients was conducted using the CFX96™ Real-Time PCR System (C1000 Touch Thermal Cycler, Bio-Rad, UAS) program for SNP detection. In brief, genomic DNA was extracted from whole blood using silica-based spin columns, such as the Qiagen purification kit (USA). Genotyping of six SNPs (FV R506Q, H1299R, Y1702 C, FII G20210 A, and MTHFR C677 T, A1298 C) was performed using ready-to-use TaqMan assays from Thermo Fisher. For each assay, 20 ng of gDNA was amplified following the manufacturer's protocol, with a final reaction volume of 20 μL in a CFX96™ Real-Time PCR System thermal cycler. Each reaction was conducted in 96-well plates, using 8 μL reactions and the TaqMan® Genotyping Master Mix, as recommended by Thermo Fisher. PCR cycling was carried out on the Applied Biosystems® 7900 Real-Time PCR System according to the manufacturer's instructions, and the data were analyzed with SDS 2.4 software. Post-amplification products were examined on the Applied Biosystems® ViiA™ 7 Real-Time PCR System, and genotype calls were manually assigned by comparing with six No Template Controls.

### DNA Purification and DNA Methylation Analysis

Genomic DNA was isolated from samples of healthy individuals and stroke patients using a DNA purification kit (Qiagen, USA), following the manufacturer's protocol. One microgram of purified DNA was treated with 1 M sodium bisulfite and 10 µL of DNA-protecting buffer in a total volume of 25 µL, using RNase- and DNase-free water. The mixture was initially denatured by incubating at 95ºC for 5 min, followed by a 7-h incubation at 50ºC to allow sodium bisulfite to convert unmethylated cytosines into uracil. To ensure complete conversion, this process was repeated three times using a thermal cycler (BIO-RAD, USA). The treated DNA was then used to amplify the promoter regions of the FV, F11, and MTHFR genes with specific primers listed in Table 1. Quantitative PCR (qRT-PCR) was performed using the QuantiTect SYBR Green PCR Kit (Qiagen, USA), with the GAPDH promoter region primer serving as a housekeeping gene for normalization. The qRT-PCR conditions were as follows: an initial denaturation at 95ºC for 5 min, followed by 35 cycles of 95ºC for 30 s, 60ºC for 30 s, and 72ºC for 30 s, with a final extension at 72ºC for 10 min to complete the amplification (Mohamed et al. 2022; Khalil et al. 2024). The methylation status within the promoter regions of the FV, F11, and MTHFR genes was determined by comparing fold changes in blood samples from stroke patients to those from healthy controls. To further assess the methylation status of the promoter regions in the specified genes, the genomic DNA was treated with a methylation-dependent restriction enzyme. A methylation-dependent enzyme, MspJI, which only cleaves the methylated cytosine in their restriction site (^m^CNNR(N)9). The digestion was carried out with 5 units of enzyme per 200 ng of DNA for 4 h at 37ºC. Post-digestion, the DNA was used for PCR amplification of the FV, F11, and MTHFR gene promoter regions, using the specific primers listed in Table 1. Conventional PCR conditions were as follows: an initial denaturation at 95ºC for 5 min, followed by 30 cycles of 95ºC for 30 s, 60ºC for 30 s, and 72ºC for 45 s. The PCR products were then resolved on a 1% agarose gel in 1X-TBE buffer and visualized under UV light (320 nm) using a gel documentation system (Elawdan et al. 2022; Guirgis et al. 2023). After amplification, methylated cytosines, cleaved by MspJI, were represented by two bands around with molecular wight of about 150 bp and 100 bp, while unmethylated fragments appeared as a single band at 250 bp.

**Table 1:** Oligonucleotides sequences used for promoter region of FV, FII, MTHFR, and GAPDH genes

Description	Primer sequences 5'−3'
FV -F	GCAAGCGCTGCCCAGGTCCT
FV–R	CCGCTTCTGTCCCTTGGCTC
FII -F	AGTGACCCAGGAGCTGACAC
FII–R	GCTGAGGAGCCAGGAACACA
MTHR-X8-F	CTGCGTTCCCCGCCCCTGC
MTHR-X8-R	TGCCCTCCAAGCAGGGGTT
GAPDH-F	ACAGTCAGCCGCATCTTCTT
GAPDH-R	CCTCCGCCCGCCCCTGCAAT

### Statistical Analysis

Hardy–Weinberg equilibrium (HWE) for each SNP was assessed using Pearson’s χ^2^ tests. This test was also employed to evaluate differences in allelic and genotypic frequencies of between patients and controls (Graffelman 2010). Delta-delta Ct analysis was employed to assess relative gene expression, represented by fold changes in steady-state mRNA levels. Ct values for the housekeeping gene, GAPDH, were utilized for normalization. A two-tailed t-test was performed to evaluate differences in the data, with *P* < 0.05 indicating statistical significance (*) and *P* < 0.01 indicating high statistical significance (**) (Maher et al. 2020; Fekry et al. 2022).

## Results

### Stroke Disorder is Associated with Elevated Levels of Homocysteine and LDL Cholesterol, as Well as Altered Levels of Vitamin B12 and Folic Acid

Demographic data of the participants, including sex, age, family history, and lifestyle, were recorded. To identify biochemical markers associated with thrombotic strokes during the acute phase, levels of homocysteine, erythrocyte sedimentation rate (ESR), and lipid parameters, such as low-density lipoprotein (LDL), were measured in peripheral venous blood samples collected after 12 h of fasting on the second day of the stroke. Additionally, the levels of vitamin B12 and folic acid were assessed in patients with thrombotic strokes to examine the potential role of vitamin B12 deficiency, which is known to influence DNA methylation processes, in the development of acute stroke in these patients. Blood samples were collected in EDTA tubes, and various lipid parameters and other blood tests were evaluated. The reference values and cut-off levels for each parameter were determined based on the recommended levels for normal, healthy individuals. The cut-off for homocysteine was set at 12 µmol/L, and for ESR, it was 20 mm/hours. For calculated LDL, normal levels were considered to be 70 mg/dL. Additionally, the cut-off levels for vitamin B12 and folic acid were defined as 203 pg/mL and 3 ng/mL, respectively. As shown in Table 2, the biochemical analysis of homocysteine levels in all patient samples revealed a significant increase, with the mean concentration of homocysteine at 25 ± 1 µmol/L (*n* = 100), compared to only two sample from healthy individuals, which showed a concentration of 13.5 µmol/L (*n* = 100). This finding indicates a strong association between elevated homocysteine levels and thrombotic strokes. Similarly, the ESR was significantly higher in-patient samples, with a mean rate of 45.5 ± 0.8 mm/h, while only six samples from healthy controls showed an elevated rate of 25 ± 4.5 mm/h. The proportion of patients and controls with LDL levels greater than 70 mg/dL were 95% and 25%, respectively (*P* < 0.01). Additionally, vitamin B12 deficiency was observed in 94 stroke patients, with a mean concentration of 90 ± 15 pg/mL, while only eight healthy control samples exhibited deficiency, with a mean concentration of 190 ± 10 pg/mL. A similarly significant deficiency in serum folate was found in 94 patient samples (1.5 ± 0.5 ng/mL), while only 10 control samples had low levels (2 ± 0.5 ng/mL). These results underscore the elevated levels of homocysteine, ESR, and LDL in stroke patients, alongside deficient levels of both vitamin B12 and serum folate.

**Table 2:** Biochemical markers serum homocysteine, ESR, LDL, vitamin B12, and serum folate in individuals with thrombotic stroke compared to healthy controls

	Homocysteine Cut-off > 12 µmol/L			ESR Cut-off > 20 mm/hours			LDL Cut-off > 70 mg/dL			Vitamin B12 Cut-off < 203 pg/mL			Serum folate Cut-off < 3 ng/mL		
	n	level	P	n	level	P	n	level	P	n	level	P	n	level	P
Co (*n* = 100)	98	10 ± 2		94	12.5 ± 0.5		75	45 ± 12		8	190 ± 10		10	2 ± 0.5	
	2	13.5 ± 0		6	25 ± 4.5		25	85 ± 10		92	255 ± 15		90	4.5 ± 0.5	
Stroke (*n* = 100)	0	0		0	0		5	65 ± 7		94	90** ± 15	0.01	94	1.5** ± 0.5	0.01
	100	25** ± 1	0.01	100	45.5** ± 0.8	0.01	95	175** ± 25	0.01	6	255 ± 25		6	5 ± 0.2	

*ERS* Erythrocyte sedimentation rate; *Co* Samples derived from healthy persons; *LDL* low-density lipoprotein

n: number of samples

*P* values ≤ 0.01 was considered as significant (**)

### The C677T Heterozygous Mutation in MTHFR is Markedly Associated with Stroke Patient-Derived Samples

**Fig. 1:**
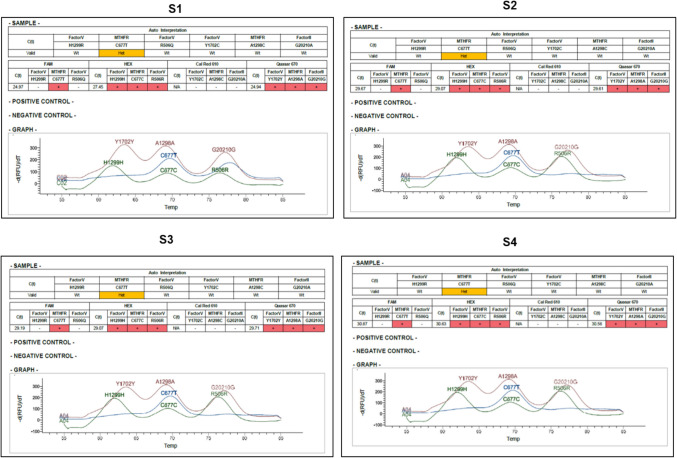
Identification of homozygous and heterozygous variants of six SNPs linked to thrombotic stroke patients. The identification of six wild-type SNPs (R506Q, H1299R, Y1702C), (G20210A), and (C677T, A1298C), along with heterozygous SNPs (R506R, H1299H, Y1702Y), (G20210G), and (C677C, A1298A) in the FV, FII, and MTHFR genes was performed using blood samples from stroke patients and healthy controls. The genotyping was conducted using the TaqMan™ SNP genotyping assay, which included specific melting curves for each SNP. The study involved 100 blood samples from stroke patients, with 100 samples from healthy individuals used as controls

**Fig. 2:**
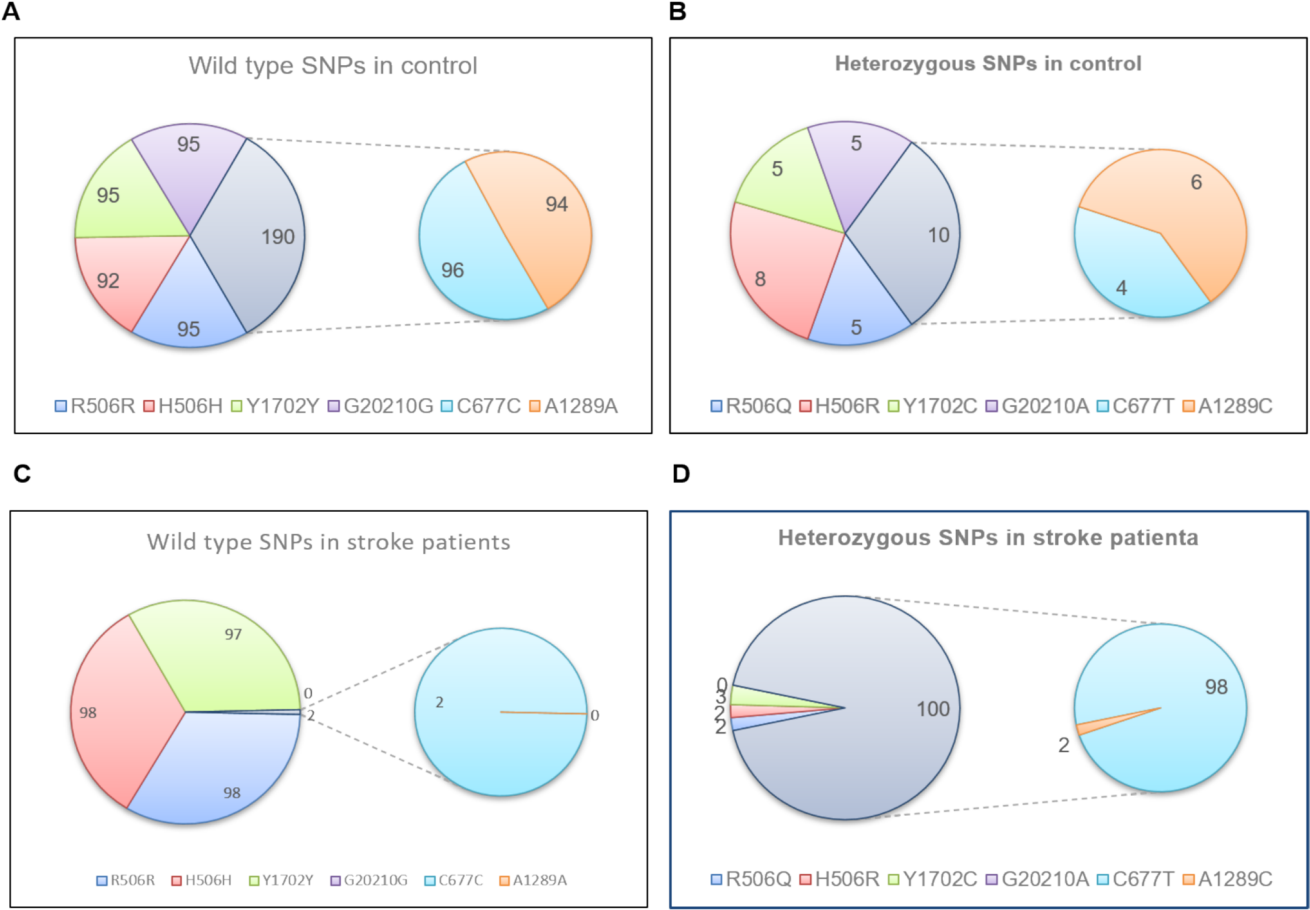
Statistical diagram illustrating the distribution of heterozygous and homozygous SNPs in stroke patient samples. (**A** and **B**) The number of wild-type and heterozygous SNPs detected in the FV, FII, and MTHFR genes from 100 blood samples of healthy individuals (Control group), with a separate, detailed chart highlighting the MTHFR gene analysis. (**C** and **D**) The number of wild-type and heterozygous SNPs identified in the FV, FII, and MTHFR genes from 100 blood samples collected from stroke patients, accompanied by a focused, standalone chart specifically illustrating the MTHFR gene analysis

All derived samples from healthy controls and stroke patients were analyzed for SNPs using the TaqMan™ SNP genotyping assay, which identified both homozygous and heterozygous SNPs in the FV, FII, and MTHFR genes. As shown in Fig. 1, the C677T heterozygous mutation in MTHFR was present in nearly all blood samples from stroke patients, as indicated by the blue melting curve. In contrast, most samples from both patients and controls exhibited homozygous SNPs, including (R506R, H1299H, Y1702Y), (G20210G), and (C677C, A1298 A) in the FV, FII, and MTHFR genes, respectively. The analysis of the identified SNPs in Fig. 2 revealed that at least 95 samples from healthy controls displayed the wild-type SNPs in the indicated genes, while 8 samples exhibited the heterozygous H1299R SNP, 5 samples showed the heterozygous Y1702C SNP, and 5 samples presented the heterozygous R506Q SNP in the FV gene. Additionally, five control samples revealed the heterozygous R506Q SNP in the FII gene, while 4 samples exhibited the heterozygous SNPs C677T and 6 samples exhibited the heterozygous SNP A1298 C in MTHFR gene (Fig. 2B). Interestingly, the wild-type SNPs for all indicated genes were detected in more than 95 samples from stroke patients, with a significant presence of the heterozygous C677T SNP in MTHFR gene (Figs. 2C and D). These findings clearly demonstrate that the MTHFR C677T heterozygous mutation is prevalent in nearly all stroke patient samples and suggest a very low likelihood of a combination between this mutation and other SNP mutations in the other indicated genes. Collectively, the data validate an association between the MTHFR C677T mutation and stroke development in the participants.

HWE was assessed for the MTHFR-C677T polymorphism, the most frequently detected SNP in patient samples, as well as for A1298C, another potentially associated MTHFR variant. Pearson’s χ^2^ test was employed to evaluate differences in both genotype and allele frequencies between stroke patients and control subjects. As presented in Table 3, the allele frequency of MTHFR-C677T showed a highly significant association with stroke cases, with a notable difference in genotype distribution between patients and controls (χ^2^ = 7.92, *P* = 0.005). These findings indicate that both genotype and allele frequencies of MTHFR-C677T are statistically significant, suggesting that the T allele is a strong risk factor for stroke in the Egyptian population.

**Table 3:** Pearson’s χ^2^ and frequency analysis of MTHFR-C677 T and A1298 C in obtained samples

SNP	A	FA	FU	B	χ^2^	P
MTHFR-C677 T	T	0.0059	0.451	C	7.92	0.005
MTHFR-A1298 C	C	0.481	0.442	A	1.52	0.21

*SNP* single-nucleotide polymorphism; *A* mutant; *B* wild-type; *FA* frequency of allele in stroke patients; *FU* frequency of allele in controls

### DNA Methylation Activity Notably Increased within the Promoter Region of MTHFR Gene

**Fig. 3:**
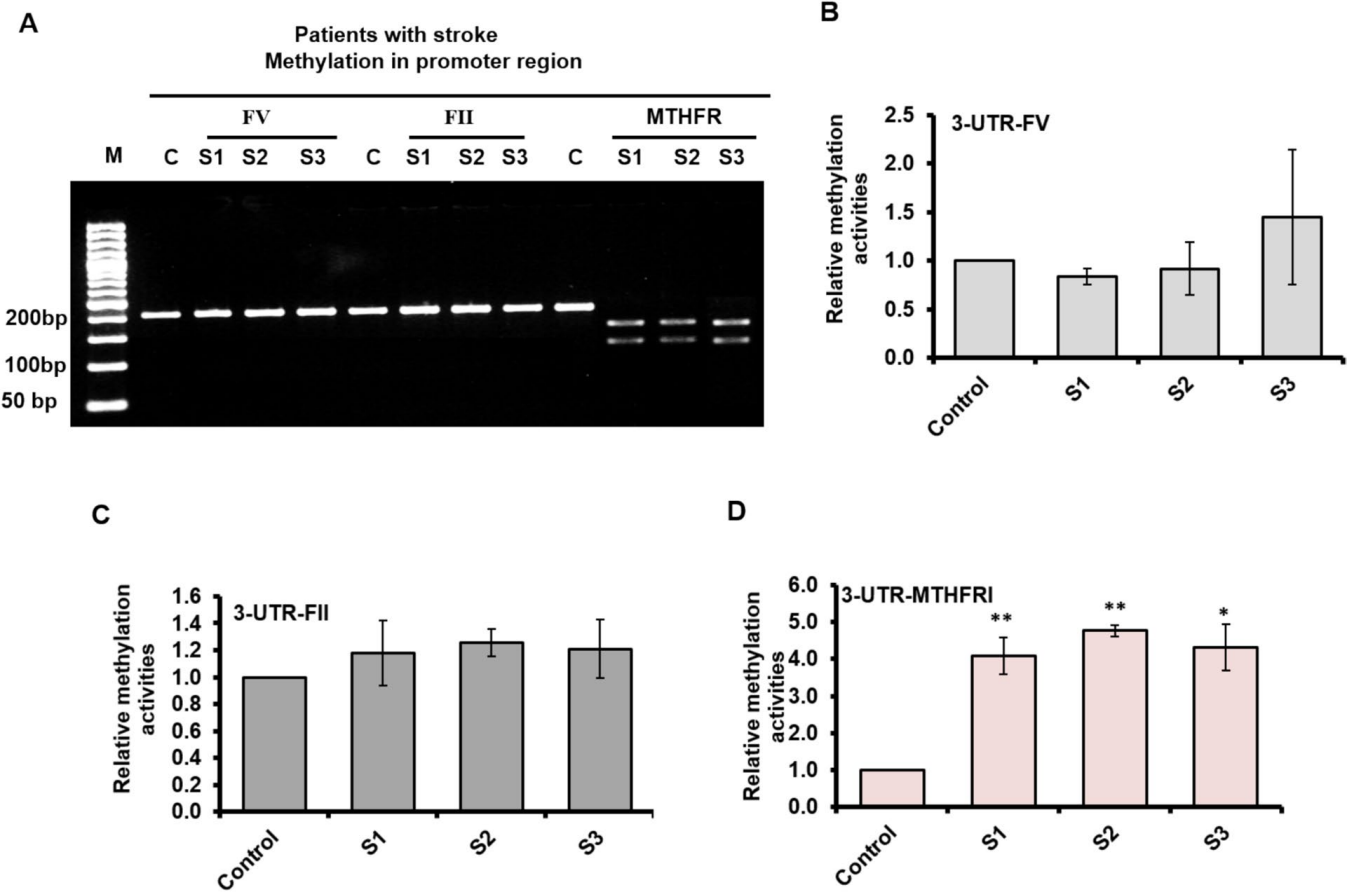
Methylation activities within the promoter regions of FV, FII, and MTHFR. (**A**) Agarose gel electrophoresis shows the MspJI-cleaved and amplified fragments of genomic DNA, which was purified from three healthy control samples and three stroke patient samples. The DNA was amplified via conventional PCR using primers specific to the promoter regions of the FV, FII, and MTHFR genes. (**B**) The relative methylation levels in sodium bisulfite-converted DNA were measured using qRT-PCR with a specific primer for the FV gene promoter region. The results are presented as fold-change in methylation activity for the three stroke patient samples compared to a sample from a healthy control. (**C**) The relative methylation levels for the FII gene promoter region were similarly assessed in sodium bisulfite-converted DNA using qRT-PCR, with the data indicating fold-change in stroke patients versus healthy controls. (**D**) The relative methylation levels for the MTHFR gene promoter region were also measured using qRT-PCR in sodium bisulfite-converted DNA, with the results shown as fold-change in stroke patients compared to healthy controls. Error bars represent the standard deviation from three replicates. A two-tailed t-test was used to assess data differences, with * indicating *P* < 0.05 for statistical significance and ** indicating *P* < 0.01 for high statistical significance. The data reflect 100 patient samples and 100 healthy control samples

To evaluate the methylation levels of cytosine nucleotides in the promoter regions of the FV, FII, and MTHFR genes, genomic DNA was treated with MspJI, an enzyme that specifically cleaves methylated cytosines at its recognition sites. The resulting fragments were then amplified via conventional PCR using gene-specific primers targeting the promoter regions of each gene. As shown in Fig. 3A and Supp. Figure 1, control samples produced a single band around 220 bp with primers for all three genes. Likewise, stroke patient samples generated the same band at the same molecular weight when primers for FV and FII were used (Supp. Figure 2). However, when primers targeting the MTHFR promoter were employed, patient samples displayed two bands at 130 bp and 90 bp (Supp. Figure 3). This outcome was attributed to MspJI cleavage at methylated cytosine sites within the MTHFR promoter region. The successful digestion indicates hypermethylation of the MTHFR promoter, while the FV and FII promoter regions exhibited normal methylation levels.

Further analysis through qRT-PCR on sodium bisulfite-converted DNA revealed no significant differences in methylation levels between stroke patients and healthy controls for the FV promoter region (Fig. 3B and Table 4), with only minimal variation observed in the FII promoter region (Fig. 3C and Table 5). In contrast, the methylation levels in the MTHFR promoter region were notably higher in stroke patients, showing a four-fold increase compared to healthy controls (Fig. 3D and Table 6). Notably, the analysis of Ct values presented in Tables 3–5 was performed using delta-delta Ct calculations, where relative methylation activity is determined by 2^ (delta-delta Ct), with the lowest conversion of cytosine by sodium bisulfate treatment reflecting higher methylation levels in the CpG islands. These findings further suggest a significant increase in the methylation of cytosine nucleotide in the MTHFR promoter region, potentially inhibiting MTHFR expression and contributing to the development of thrombotic strokes in patients.

**Table 4:** Delta-delta Ct analysis indicated the fold change in methylation activity within the promoter region of FV

	GAPDH		FV		Δ Ct1	Δ Ct2	Δ- Δ Ct1	Δ- Δ Ct2	Fold change Ct1	Fold change Ct2	Mean Fold change	SD	*P* Value
	Mean Ct1	Mean Ct2	Mean Ct1	Mean Ct2									
**Control**	18.44	18.71	29.87	28.37	11.44	1.2	0.00	0.00	1.00	1.00	1.00	0.00	
**S1**	19.46	18.70	30.53	28.20	11.08	1.6	−0.36	−0.15	0.78	0.90	0.84	0.08	0.11
**S2**	18.43	18.49	29.40	28.30	10.97	1.9	−0.47	0.15	0.72	1.11	0.92	0.27	0.71
**S3**	18.95	18.46	29.96	27.53	11.01	1.8	−0.07	−0.43	0.96	1.94	1.45	0.70	0.46

**Table 5:** Delta-delta Ct analysis indicated the fold change in methylation activity within the promoter region of FII

	GAPDH		FII		Δ Ct1	Δ Ct2	Δ- Δ Ct1	Δ- Δ Ct2	Fold change Ct1	Fold change Ct2	Mean Fold change	SD	*P* Value
	Mean Ct1	Mean Ct2	Mean Ct1	Mean Ct2									
**Control**	18.44	18.71	29.20	28.10	10.76	9.39	0.00	0.00	1.00	1.00	1.00	0.00	
**S1**	19.46	18.70	30.23	28.52	10.78	9.82	0.01	0.43	1.01	1.35	1.18	0.24	0.41
**S2**	18.43	18.49	29.60	28.13	11.17	9.64	0.41	0.25	1.33	1.19	1.26	0.10	0.07
**S3**	18.95	18.46	30.16	27.93	11.21	9.47	0.45	0.08	1.36	1.06	1.21	0.22	0.30

**Table 6:** Delta-delta Ct analysis indicated the fold change in methylation activity within the promoter region of MTHFR

	GAPDH		MTHFR		Δ Ct1	Δ Ct2	Δ- Δ Ct1	Δ- Δ Ct2	Fold change Ct1	Fold change Ct2	Mean Fold change	SD	P Value
	Mean Ct1	Mean Ct2	Mean Ct1	Mean Ct2									
**Control**	18.44	18.71	29.13	28.23	10.70	9.52	0.00	0.00	1.00	1.00	1.00	0.00	
**S1**	19.46	18.70	32.30	30.12	12.85	11.42	2.15	1.90	4.43	3.73	4.08**	0.49	0.01
**S2**	18.43	18.49	31.34	30.30	12.91	11.81	2.22	2.28	4.65	4.87	4.76**	0.16	0.01
**S3**	18.95	18.46	31.60	30.23	12.65	11.78	1.95	2.25	3.87	4.76	4.32*	0.63	0.02

## Discussion

In this study, we examined six heterozygous SNPs related to FV, FII, and MTHFR in blood samples from stroke patients, comparing them to samples from healthy individuals. Additionally, we explored the biological relationship between elevated homocysteine and LDL levels, as well as the reduced levels of vitamin B12 and folic acid, in the development of thrombotic stroke. This led us to consider the possible involvement of hypermethylation in the promoter regions of MTHFR, which plays a crucial role in regulating blood homocysteine levels in conjunction with vitamin B12 and folic acid. Our findings notably confirmed the presence of the heterozygous MTHFR-C677T mutation in more than 95% of the stroke patient samples, while other heterozygous SNPs were rarely observed. Furthermore, we observed a significant increase in methylation activity in the MTHFR promoter region in stroke patients compared to the FV and FII genes, both in control and stroke-derived samples. These results highlight the potential defect in MTHFR gene expression in stroke patients, which could explain the elevated homocysteine levels and the development of thrombotic stroke. The homocysteine amino acid in the blood is commonly used to detect deficiencies in vitamins B12 and folic acids. Elevated homocysteine levels may signal an increased risk for cardiovascular diseases and stroke. In newborns, it can also help diagnose homocystinuria, a rare genetic disorder (Walter et al. 2015). Dyslipidemia is a significant risk factor for atherosclerosis-related ischemic stroke. Stroke patients often have elevated total cholesterol (TC) and LDL levels, along with reduced high-density lipoprotein (HDL) levels. This trend is concerning, particularly with the rising incidence of ischemic stroke among both young and elderly populations (Ciplak et al. 2023). The MTHFR protein plays an essential role in processing folate to break down homocysteine. Under normal conditions, the body rapidly processes homocysteine, keeping its blood levels low. However, mutations in the MTHFR gene, such as C677T and A1298C, reduce the effectiveness of the MTHFR protein, leading to higher homocysteine levels. This increase in homocysteine can elevate the risk of blood clots, heart disease, neural tube defects, and stroke.

Methylation, which involves the addition of a methyl group to activate a molecule, is crucial for the proper functioning of metabolic pathways and the efficient activity of enzymes. This process plays a significant role in various biochemical reactions that regulate essential bodily functions, such as gene expression (Menezo et al. 2020). Essentially, methylation acts like an on/off switch, allowing molecules and processes to be activated or deactivated to perform necessary functions or trigger reactions (Mattei et al. 2022). A mutation in the MTHFR gene can lead to a defective or insufficient MTHFR enzyme, which can disrupt methylation. When methylation is impaired, it may result in metabolic disorders and overall poorer health. DNA, RNA, proteins, and lipids all require methylation to function properly. A malfunctioning MTHFR enzyme can reduce methylation, leading to inadequate folate activity and poor folate conversion (Jiménez et al. 2018). Additionally, low folate means fewer methyl groups are available to methylate other molecules, which can cause a buildup of toxic substances and heavy metals (Cao et al. 2022). Moreover, the C667 T homozygous mutation in the MTHFR gene is associated with elevated homocysteine levels. If homocysteine cannot be converted into methionine, it becomes harmful to the body, and MTHFR mutations may lead to decreased ATP production (Liew and Gupta 2015). Consequently, we proposed for the first time that the hypermethylation of the MTHFR gene, combined with the presence of the C667T homozygous mutation and elevated homocysteine levels increases the risk of thrombotic stroke. This conclusion aligns with a study that suggested a possible association between the heterozygous C677T polymorphism in the MTHFR gene, elevated homocysteine levels, and gene, environment interactions contributing to early neurological deterioration (END) susceptibility in Chinese patients (Zhou et al. 2024). Furthermore, evidence suggests that the MTHFR T677T and A1298A genotypes are significantly linked to an increased risk of hemorrhagic stroke in Turkish patients. In contrast, the C1298C genotype and the compound C677C/C1298C genotype are significantly associated with ischemic stroke (Sazci et al. 2006).

Notably, elevated homocysteine, a condition known as hyperhomocysteinemia, is associated with risk factors for neurovascular diseases like stroke and hydrocephalus. Hyperhomocysteinemia contributes to neuroinflammation, oxidative stress, endothelial dysfunction, and disruption of the blood–brain barrier, which ultimately leads to neurodegeneration (Ortiz-Salguero et al. 2025). These findings highlight hyperhomocysteinemia as both a biomarker and a potential therapeutic target for vascular-related neurological disorders. Recent research has indicated that polyphenols may help reduce hyperhomocysteinemia and offer protection against neurodegenerative diseases (Jalouli et al. 2025). Similarly, addressing epigenetic changes through DNA hypermethylation has been suggested as a novel therapeutic strategy for treating ischemic stroke and brain injury following a stroke (Choi et al. 2022). In line with our hypothesis, early evidence underscores the significant role of DNA methylation in post-stroke brain damage and points to widespread DNA hypermethylation following brain ischemia as a contributing factor to brain damage (Endres et al. 2000; Jung et al. 2002).

## Conclusion

This study aimed to investigate the potential link between specific SNPs and thrombotic strokes in Egyptian patients, as well as the impact of DNA methylation in the promoter regions of genes associated with these SNPs. The study involved 100 adult patients who were consecutively admitted to the International Medical Center, all of whom were diagnosed with acute ischemic stroke. These patients were compared to age-matched controls (± 3 years). The molecular analysis focused on six thrombosis-related SNPs: FV (R506Q, H1299R, Y1702C), FII (G20210A), and MTHFR (C677T, A1298C), using blood samples from both stroke patients and healthy controls. DNA methylation in the promoter regions of the FV, FII, and MTHFR genes was assessed through a sodium bisulfite conversion method and genomic DNA digestion with the methylation-dependent restriction enzyme MspJI, using specific primers for the promoter regions of these genes in all derived samples. The biochemical analysis of the samples revealed higher levels of homocysteine, ESR, and LDL in stroke patients, alongside lower levels of vitamin B12 and serum folate. The SNP analysis, performed using the TaqMan™ SNP genotyping assay, identified homozygous SNPs in the FV, FII, and MTHFR genes. The results indicated that nearly all stroke patient samples contained the MTHFR C677T heterozygous mutation, with a very low likelihood of this mutation accompanied with mutations in the other genes examined. Methylation analysis of the promoter regions of these genes showed hypermethylation in the MTHFR promoter region, while methylation levels in the FV and FII promoter regions were normal. Increased methylation of the cytosine nucleotide in the MTHFR promoter region was observed, potentially inhibiting MTHFR expression and contributing to the development of thrombotic strokes in these patients. Overall, the findings suggest an association between the MTHFR C677T mutation, hypermethylation in its promoter region, and stroke development in the study participants.

## Data Availability

No datasets were generated or analysed during the current study.
